# Relationship Between Chinese Adolescents’ Sleep Status and Problem Behaviors: The Mediating Role of Mental Health

**DOI:** 10.3389/fpsyg.2021.689201

**Published:** 2021-09-14

**Authors:** Ying Li, Shuhang Zhao, Weidong Li, Hongyan Liu

**Affiliations:** ^1^School of Public Administration, Zhongnan University of Economics and Law, Wuhan, China; ^2^Center for Experimental Economics in Education (CEEE), Shaanxi Normal University, Xi’an, China; ^3^School of Philosophy and Government, Shaanxi Normal University, Xi’an, China; ^4^School of Economics, Northwest University of Political Science and Law, Xi’an, China

**Keywords:** adolescent, problem behavior, sleep status, mental health, mediating effect

## Abstract

Adolescents’ problem behaviors constitute a critical indicator of crime, and they play an important role in the growth and development of adolescents and social stability. Using the 2014–2015 school year follow-up data from the China Education Panel Survey, this study investigated the relationship between sleep time, sleep disorders, and adolescents’ problem behaviors. Further, we analyzed the mediating effect of mental health status, a key factor influencing adolescent growth and development. The results showed that the frequency of problem behaviors among adolescents was significantly and positively associated with sleep disorders and short sleep time. In addition, mental health status is a channel through which sleep time and sleep disorders affect adolescents’ problem behaviors.

## Introduction

To rejuvenate the Chinese nation, adolescents are to be considered an essential reserve force. Social behaviors of adolescents are closely linked to their personal growth and directly affect the nation’s future. From a life-course perspective, adolescence is not only a critical period of personal physical and mental growth, but also a period of increased problem and antisocial behaviors caused by a lack of healthy physical and mental development (Feldman and Elliot, 1990; Moffitt, 2006). According to Jessor (1987), problem behavior is behavior that departs from the norms—both social and legal—of a greater society. In addition, it has been socially disapproved by the institutions of authority, and it tends to elicit some form of social control response, whether mild reproof, social rejection, or even incarceration. Based on the research objects of this study, problem behavior, which is also known as “deviant behavior,” “deviance,” or “delinquency behavior,” has been defined in this study as behaviors that are more common to middle school students, deviate from school rules and regulations, and have a negative impact on daily life, study, and social security. According to the China Family Panel Studies in 2010, a total of 75.22% of adolescents exhibited deviant behaviors (Liu, 2016). A survey in Beijing showed that over 60% of adolescents had more than two forms of problem behaviors, of which 59.6% attending ordinary middle and technical schools smoked and drank alcohol, and 46.8% fought and abused others (Research group of the Beijing Municipal Commission of the Chinese Communist Youth League, 2015). In addition, studies have shown that adolescent problem behaviors can affect normal study and life (Youth Deviant Behavior Research Project of the Zhejiang Provincial Commission of the Chinese Communist Youth League, 2013). Moreover, if the problem behaviors are not effectively restrained, more severe consequences may occur, such as illegal or criminal behaviors in adolescence and even adulthood (Yao, 2012; Youth Deviant Behavior Research Project of the Zhejiang Provincial Commission of the Chinese Communist Youth League, 2013). Therefore, reinforcing the analysis of adolescents’ problem behavior, investigating its mechanism of influence, and delivering targeted preventive measures are of great importance to the growth of individuals and society.

Studies have shown that the academic status (Jessor et al., 1995), parental involvement (Wu and Zhang, 2018), and deviant peers (Hou et al., 2017) are all significant factors influencing adolescents’ problem behaviors. Furthermore, sleep has a major effect on the problem behaviors of adolescents. Short sleep time or sleep problems will increase the frequency of adolescents’ problem behaviors to varying degrees (Vail-Smith et al., 2009; Barnes and Meldrum, 2014). Sleeping provides opportunities for the human central nervous system to recover and regenerate, and sleeping is quite important for the development of people of all ages, especially adolescents. Insufficient sleep time may damage the prefrontal cortex function; weaken the inhibition of self-aggression; and make it easier for people to be impulsive, angry, and not conducive to decision making (Dahl, 2006; McKenna et al., 2007). Moreover, long-term sleep disorders disrupt the adolescents’ biological clock, making the cerebral cortex tense for a long time. Consequentially, it will not effectively regulate the nervous system (Wu and Song, 2011), which can also trigger adolescents’ problem behaviors.

Studies have shown that poor mental status is a major cause of problem behaviors (Wu, 2000), while sleep disorders and inadequate sleep time have been found to have a detrimental effect on mental health status (Fredriksen et al., 2004; Alfano et al., 2009). The relationship between sleep status (sleep time and sleep disorders) and mental health is even stronger during adolescence (Gregory and Sadeh, 2012). A study conducted in Chicago, the United States, that included 2,259 participants aged 11–14 years for instance found that junior high school students who slept less showed more depressive symptoms (Fredriksen et al., 2004). In a study by Alfano et al. (2009) conducted in Louisiana, the United States, the existence of sleep disorders (such as nightmares, sleepwalking, and other sleep problems) was found to lead to a deterioration of the mental state of adolescents, and they were more prone to anxiety and depression. Therefore, to a certain extent, sleep time and sleep disorders may affect adolescents’ mental health and have a particular impact on their problem behaviors.

In summary, while many studies have shown that sleep time and sleep disorders are significantly linked to adolescents’ problem behaviors, the mediating effect of their mental status has not been analyzed, to the best of our knowledge. In addition, there is a lack of research that employs nationally representative evidence. With this in mind, the 2014–2015 China Education Panel Survey (CEPS) data were used in this study to investigate the relationship between the sleep status (sleep time, sleep disorders) of Chinese adolescents and their problem behaviors, and then to assess and discuss the mediating effect of adolescents’ mental health status.

## Literature Review

### Research on the Relationship Between Adolescents’ Sleep and Problem Behaviors

Sleep guarantees normal growth and development in adolescents (Minges and Redeker, 2016). In contrast, lack of sleep time and the presence of sleep disorders may increase the frequency of adolescents’ problem behaviors (Vail-Smith et al., 2009; Barnes and Meldrum, 2014). The influence of sleep on problem behaviors from sleep time and sleep disorders (such as difficulty falling asleep and lethargy) has been discussed in past research.

In terms of the impact of sleep time on problem behaviors, Peach and Gaultney (2013) found that lack of sleep increased the frequency of behaviors such as fighting, stealing, and damaging other people’s property. Furthermore, Barnes and Meldrum (2014) examined 289 adolescent monozygotic (MZ) twin pairs, after excluding genetic factors, and found that lack of sleep increases the occurrence of both violent and non-violent delinquency (including behaviors such as smoking and drinking) among adolescents. The same conclusion was obtained by studies using other data (O’Brien and Mindell, 2005; Yen et al., 2010). Some scholars have also researched specific types of problem behaviors. In a study by Yen et al. (2010), which included a total of 8,319 adolescents from southern Taiwan, adolescents with a total sleep time of < 6 h were defined as “short sleepers,” and those with a total sleep time >8 h were defined as “long sleepers.” Others were defined as “average sleepers.” Short sleepers were found to have substantially higher rates of truancy, use of illicit drugs, and violence against others than average sleepers. A literature review of the relationship between sleep and aggressive behavior was carried out by Kamphuis et al. (2012), who found that lack of sleep increased problematic daytime behavior, such as aggression and behavior problems. However, there is no clear linear relationship between sleep time and problem behavior, and sleep time is not considered as the more, the better. Studies also found that the proportion of long sleepers (> 8 h) who were aggressive, used illicit drugs, and had more tattoos was significantly higher than that of average sleepers (Yen et al., 2010).

However, adolescents’ sleep problems may not be fully reflected by their sleep time, whereas sleep disorders^1^ are a common component of sleep problems. For instance, a survey of 859 undergraduates from a public university in the southeastern United States found that the most common problems among adolescents were general morning tiredness (82%) and insomnia (28%) (Vail-Smith et al., 2009). Regarding the prevalence of sleep disorders, surveys from various countries have shown that 20%–25% of adolescents reported excessive daytime sleepiness (Pagel et al., 2007; Roehrs et al., 2011), and adolescents with insomnia symptoms accounted for 19.3%–37.1% (Vail-Smith et al., 2009; Luo et al., 2012; Jing et al., 2016; Chahoud et al., 2017; Li et al., 2018). Similar to insufficient sleep time, there is a greater occurrence of problem behaviors among adolescents with sleep disorders. For example, a study of 4,353 US adolescents from the National Longitudinal Study of Adolescent Health (Add Health) showed that adolescents with insomnia symptoms smoked more than their peers (Catrett and Gaultney, 2009). Other studies have found that severe sleep disorders may have various adverse consequences, including smoking and alcohol use (Vail-Smith et al., 2009). Research has also found that sleep disorders not only affect adolescents themselves, but also affect getting along with others. A study of 4,175 youths aged 11–17 from a large metropolitan area in the United States found that adolescents with insomnia disorders, including difficulty in falling asleep and easy to wake up, were more likely to show interpersonal problems (Roberts et al., 2008). A study of middle school students in England who were aged 14–16 years also found that teenagers’ daytime sleepiness would increase problem behaviors such as fighting (Raine and Venables, 2017).

### The Relationship Between Sleep, Mental Health Status, and Adolescents’ Problem Behaviors

Sleep time and sleep disorders affect adolescents’ problem behaviors not only directly, but also indirectly by influencing their mental health status. Studies found that adequate sleep time positively affects the mental health of adolescents (Jamieson et al., 2019), whereas insufficient sleep time and sleep disorders can worsen it (Kahn-Greene et al., 2006; Alfano et al., 2009; Gregory and Sadeh, 2012; Owens, 2014). According to existing research, inadequate sleep time may increase anger, irritability, and depression (Kamphuis et al., 2012). In addition, adolescents with sleep disorders are more likely to be anxious, provocative, lethargic, and impulsive (Kahn-Greene et al., 2006; Roberts et al., 2008; Alfano et al., 2009; Gregory and Sadeh, 2012), and they exhibit more anger, frustration, and nervousness (Morrison et al., 1992; Lund et al., 2010). Moreover, mental health status has been found to affect adolescents’ problem behaviors, and poor mental status is a significant factor in triggering them (Wu, 2000). Chen and Zhong (2012) conducted a study that included 1,411 second-year students in Guangzhou and found that negative emotions such as nervousness, desperation, depression, or feeling worthless do have a significant effect on adolescents’ problem behaviors such as smoking, drinking, fighting, and Internet use for more than 5 h a day, indicating addiction. Thus, mental health status can play a key mediating role in the impact of sleep on problem behavior.

In addition, the existing studies on the relationship between sleep time, sleep disorders, and adolescents’ problem behaviors are primarily concentrated in Western countries, and there are few Chinese studies on this topic. On the one hand, a global meta-analysis regarding sleep habits among adolescents aged 11–18 years found that Asian adolescents went to bed substantially later than their peers in North America and Europe. In addition, they had less overall sleep time and a higher rate of daytime sleepiness (Gradisar et al., 2011). Therefore, it is meaningful to conduct research on the relationship between sleep status and problem behaviors in Asian and particularly Chinese adolescents. The path toward how sleep affects adolescents’ problem behaviors still needs to be thoroughly studied. Thus, we examined the relationship between Chinese adolescents’ sleep time, sleep disorders, and their problem behaviors. Further, we also analyzed the mediating effect of adolescents’ mental status by performing an Ordinary Least Squares (OLS) regression and mediation analysis with data from CEPS during the 2014–2015 school year.

## Data and Methods

### Ethics

The studies involving human participants were reviewed and approved by the Ethics Committee of the Renmin University of China. The participants’ legal guardian/next of kin provided written informed consent for their participation in this study.

### Data

Data used in the current study come from the tracking data of the CEPS during the 2014–2015 school year. The CEPS was designed and implemented by the National Survey Research Center at the Renmin University of China. A multi-stage probability-proportional-to-size sampling methodology was adopted for the survey, while random sampling focused on population mobility and education. A total of 438 classes in 112 schools from 28 county-level units across the country, which is considered a nationally representative sample, were included in the final sample. A total of 10,279 seventh-grade students during the 2013–2014 baseline survey were monitored in the follow-up survey for the 2014–2015 school year. A total of 830 students were missing during follow-up because of school transfers or dropout. The follow-up success rate was 91.9%. The final sample size of the tracking data for the 2014–2015 school year was 9,449. The same questionnaires as for the baseline survey were used in the follow-up interview, namely the student, parent, head teacher, core subject teacher, and school administrator questionnaires. Most of the variables used in this paper come from the student questionnaire in the 2014–2015 follow-up survey, while the basic student information (such as gender and ethnicity) comes from the student questionnaire used in the baseline survey for 2013–2014. The final analysis included a total of 7,879 participants after extracting the missing values of the variables.

### Variables

#### Dependent Variable: Adolescents’ Problem Behaviors

Human beings aged 10–19 years are defined as adolescents internationally (World Health Organization, 2006). Based on the specificity of research objects, this study defines problem behaviors as those behaviors that are more common to middle school students, deviate from school rules and regulations, and have a negative impact on daily life, study, and social security, including swearing, quarreling, fighting, bullying vulnerable students, skipping class/truancy, copying homework or cheating on exams, smoking/drinking, and going to Internet cafes/game halls. In the “Social Behavior Development” section of the CEPS questionnaire, this variable is measured by the question, “Did you have the following behaviors in the past year: swearing; quarreling; fighting; bullying vulnerable students; skipping classes/truancy; copying homework/cheating on exams; smoking/drinking; going to Internet cafes/game halls?” The answer to each question is selected from five possible responses: “never,” “occasionally,” “sometimes,” “often,” or “always.” The values are assigned to 1, 2, 3, 4, and 5, respectively. The total score was determined by calculating the score on the eight questions. The higher the score, the greater the frequency of problem behaviors among adolescents. After that, according to whether the object of the problem behaviors was oneself or others, they were divided into two categories: problem behaviors that threaten others and problem behaviors that threaten oneself. The first four items belong to problem behaviors that threaten others, and the rest of the items belong to problem behaviors that threaten oneself.

#### Independent Variable: Sleep Time and Sleep Disorder

This variable is measured using the questions in the “Physical and mental health” section of the CEPS questionnaire: “How long do you usually sleep at night?” and “Usually, do you have the following sleep problems?” Students who slept < 8 h at night were categorized as short sleepers, and those who slept for ≥8 h were categorized as long sleepers. Students who chose any one or more of the following problems were considered to have sleep disorders: difficulty falling asleep, easy to wake up, fatigue after waking up, sleepwalking, dreaminess, somnambulism, snoring, lethargy, and tooth grinding. Only when they did not choose any sleep problems it was considered that there was no sleep disorder.

#### Mediating Variable: Mental Health Status

This variable was measured by the questions in the “Physical and mental health” section of the CEPS questionnaire: “During the past 7 days, did you have the following feelings: depressed; depressed too much to concentrate on doing things; unhappy; life is meaningless; unable motivate to do things; sad; nervous; worrying too much; foreboding that bad thing will happen; too much energy and not paying attention in class?” The answer to each question was selected from five possible responses: “never,” “occasionally,” “sometimes,” “often,” or “always.” The values were assigned to 1, 2, 3, 4, and 5, respectively. The total score was determined by calculating the score on the questions. The higher the score, the worse the mental health status of adolescents.

The control variables included individual student characteristics and family characteristics such as students’ sex, ethnicity, Hukou type, school boarding status, cognitive ability,^2^ peer misconduct,^3^ parents’ marital status, academic pressure (language, math, English), self-evaluation of the family’s economic status, their parents’ academic expectations, whether their family members smoked,^4^ whether they often eat junk food, and so forth.

### Methods

Through descriptive statistics, this study first described the basic characteristics of the sample adolescent individuals and their families and then established the following multiple linear regression model analysis to examine the relationship between sleep time, sleep disorders, and adolescents’ problem behaviors.


(1) $${\text{Y}}_{i}=\beta_{0}+\beta_{1,,}*\text{Sleep}+\beta_{2,,}*\text{Student}+\beta_{,3,}*\text{Family}+\varepsilon_{i}$$

$${\text{Y}}_{i}=\beta_{,0,,}+\beta_{,1}*\text{Sleep}+\beta_{,2}*\text{Student}+\beta_{,3}*\text{Family}+$$

(2) $$\beta_{,4}*\text{Mental}+\varepsilon_{i}.$$

**Y_i_** represents the students’ problem behavior scores. **Sleep** represents adolescents’ sleep time and sleep disorder variables. **Student** represents individual student characteristic variables, including sex, ethnicity, and cognitive ability. **Family** represents the adolescent’s family characteristic variables, specifically whether he or she is an only child, whether the parents are married, and so forth. **Mental** health stands for student’s mental health score. ε**_i_** is the residual item.

**FIGURE 1:**
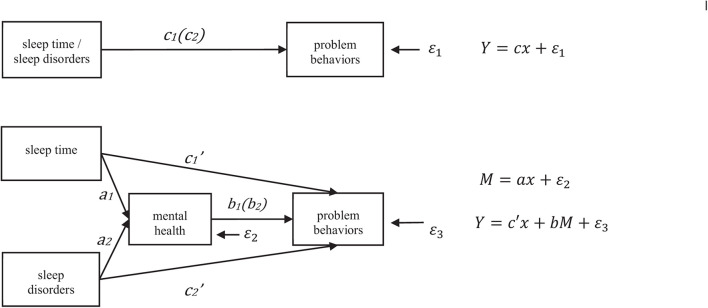
Mediating effect model.

Equation (1) represents a model without the adolescents’ mental health variables, and Equation (2) represents a model that includes them. This study determined whether to use adolescents’ mental health as a mediating variable for mediating effect analysis (Figure 1) after comparing the results of the two models and testing through the seemingly uncorrelated regression estimation method.

When identifying the causal relationship, a randomized controlled trial has always been considered the gold standard for evaluating the impact. However, studies based on cross-sectional data cannot be randomized. Therefore, we can artificially generate many pairs of “twins” in cross-sectional data using certain statistical methods so that the screened and matched treatment and control groups are comparable in some important individual characteristics (i.e., potential confounding factors). The propensity score matching (PSM) method can somewhat alleviate the endogeneity problem and the estimation bias caused by the selection bias. Specifically, short sleepers and adolescents without sleep disorders were considered as the control group, while long sleepers and adolescents with sleep disorders were considered as the treatment group in the study. There are also many matching methods for researchers to choose from. Nearest-neighbor-matching, radius-matching, and kernel-matching methods were used in the study. For more technical information about propensity score methods, refer to Austin (2011). Regarding the choice of the mediating effect test method, the causal steps approach proposed by Baron and Kenny (1986) will cancel the direct and indirect effects (i.e., suppression effect) by ignoring some specific mediating effects (MacKinnon et al., 2000). Therefore, the more widely used product of coefficients approach (*H*_0_:*ab* = 0) was used in this study to test the mediating effect. Among the commonly used estimation methods, the Sobel test requires a^∗^b to follow a normal distribution, which is difficult to achieve, while bootstrap obtains a more accurate standard error through sampling with replacement, which has higher statistical power. The bootstrap method is recognized as a method to replace the Sobel method to test the product of coefficients directly (Wen and Ye, 2014). Therefore, this study used the bootstrap estimation method to evaluate the mediating effect. Furthermore, statistical software Stata 15.1 was used for data processing, descriptive statistics, multiple linear regression analysis, and mediating effect estimation.

## Results

### Descriptive Results

According to the descriptive statistics shown in Table 1, the scores of adolescents’ problem behaviors were generally low, indicating that the frequency of adolescents’ problem behaviors was low as well. Overall, most adolescents (62.58%) sleep longer, while almost half (44.40%) of adolescents have sleep disorders.

**TABLE 1:** Descriptive results.

**Variables**	**Total sample**		**Male**		**Female**		***P-* value**	**An only child**		**Non-only child**		***P-* value**
	**Sample size**	**Mean/%**	**Sample size**	**Mean/%**	**Sample size**	**Mean/%**		**Sample size**	**Mean/%**	**Sample size**	**Mean/%**	
***Dependent variable***												
Total problem behaviors	7879	11.37	4017	12.08	3862	10.63	0.000	3538	10.94	4341	11.71	0.000
Problem behaviors threaten others	7879	6.93	4017	6.93	3862	6.10	0.000	3538	6.29	4341	6.71	0.000
Problem behaviors threaten themselves	7879	4.84	4017	5.15	3862	4.53	0.000	3538	4.65	4341	5.00	0.000
***Independent variable***												
Sleep time							0.000					0.000
Short sleeper	2948	37.42	1337	33.28	1611	41.71		1478	41.78	1470	33.86	
Long sleeper	4931	62.58	2680	66.72	2251	58.29		2060	58.22	2871	66.14	
Sleep disorders							0.000					0.001
Yes	3498	44.40	1636	40.73	1862	48.21		1499	42.37	1999	46.05	
No	4381	55.60	2381	59.27	2000	51.79		2039	57.63	2342	53.95	
***Mediating variable***												
Mental health status	7879	–0.01	4017	–0.04	3862	0.01	0.000	3538	–0.10	4341	0.05	0.000
***Control variables***												
**Individual characteristics**												
Gender							–					0.000
Male	4017	50.98	–	–	–	–		1941	54.86	2076	47.82	
Female	3862	49.02	–	–	–	–		1597	45.14	2265	52.18	
Ethnicity							0.072					0.000
Han	7242	91.92	3714	92.46	3528	91.35		3345	94.54	3897	89.77	
Others	637	8.08	303	7.54	334	8.65		193	5.46	444	10.23	
Hukou type							0.081					0.000
Rural Hukou	4155	52.74	2157	53.70	1998	51.73		1112	31.43	3043	70.10	
Non-rural Hukou	3724	47.26	1860	46.30	1864	48.27		2426	68.57	1298	29.90	
Resident student							0.866					0.000
Yes	2396	30.41	1225	30.50	1171	30.32		522	14.75	1874	43.17	
No	5483	69.59	2792	69.50	2691	69.68		3016	85.25	2467	56.83	
Cognitive ability	7879	0.34	4017	0.31	3862	0.38	0.000	3538	0.50	4341	0.21	0.000
Problem behaviors of peers	7879	5.76	4017	6.16	3862	5.35	0.000	3538	5.64	4341	5.86	0.000
Academic pressure of Chinese							0.000					0.000
Yes	2369	30.07	1466	36.49	903	23.38		931	26.31	1438	33.13	
No	5510	69.93	2551	63.51	2959	76.62		2607	73.69	2903	66.87	
Academic pressure of math							0.000					0.000
Yes	4098	52.01	1947	48.47	2151	55.70		1596	45.11	2502	57.64	
No	3781	47.99	2070	51.53	1711	44.30		1942	54.89	1839	42.36	
Academic pressure of English							0.000					0.000
Yes	4257	54.03	2551	63.51	1706	44.17		1560	44.09	2697	62.13	
No	3622	45.97	1466	36.49	2156	55.83		1978	55.91	1644	37.87	
Often eat junk food							0.000					0.002
Yes	4683	59.44	2270	56.51	2413	62.48		2170	61.33	2513	57.89	
No	3196	40.56	1747	43.49	1449	37.52		1368	38.67	1828	42.11	
Often drink sugary or carbonated beverages							0.000					0.000
Yes	5031	63.85	2642	65.77	2389	61.86		2342	66.20	2689	61.94	
No	2848	36.15	1375	34.23	1473	38.14		1196	33.80	1652	38.06	
**Family characteristics**												
Economic status							0.000					0.000
Economic hardship	1168	14.82	627	15.61	541	14.01		259	7.32	909	20.94	
Middle class	5786	73.44	2863	71.27	2923	75.69		2719	76.85	3067	70.65	
Wealthy	925	11.74	527	13.12	398	10.31		560	15.83	365	8.41	
Parents married							0.007					0.000
Yes	7236	91.84	3722	92.66	3514	90.99		3173	89.68	4063	93.60	
No	643	8.16	295	7.34	348	9.01		365	10.32	278	6.40	
An only child							0.000					–
Yes	3538	44.90	1941	48.32	1597	41.35		–	–	–	–	
No	4341	55.10	2076	51.68	2265	58.65		–	–	–	–	
Education expectation							0.000					0.000
High school and below	1242	15.76	796	19.82	446	11.55		408	11.53	834	19.21	
Universities or colleges graduates	4304	54.63	2060	51.28	2244	58.10		1919	54.24	2385	54.94	
Postgraduate and above	2022	25.66	966	24.05	1056	27.34		1075	30.38	947	21.82	
Not to matter	311	3.95	195	4.85	116	3.00		136	3.84	175	4.03	
Family members smoke							0.804					0.000
Yes	4865	61.75	2475	61.61	2390	61.89		2074	58.62	2791	64.29	
No	3014	38.25	1542	38.39	1472	38.11		1464	41.38	1550	35.71	

In terms of the individual student characteristics, among the sample, the gender distribution of boys and girls was reasonably balanced; the proportion of rural Hukou type was 52.74%; the proportion of non-resident students (69.59%) was higher than that of resident students (30.41%). The pressure to learn English was considerably greater than that of Chinese; the overall living habits of adolescents were poor, and the proportions of samples that often eat junk food and drink sugary or carbonated beverages were 59.44% and 63.85%, respectively.

In terms of family characteristics, 44.90% of adolescents were the only children; 73.44% of adolescents evaluated the family’s economic status as moderate, and 91.84% of adolescents’ parents were married. In terms of parental academic expectations for teenagers, most parents hoped that their child would obtain a college/undergraduate degree or higher. In terms of family environment, up to 61.75% of adolescents reported that family members smoked together often.

### OLS Regression Results

Table 2 shows that sleep time was significantly and negatively correlated with adolescents’ problem behaviors after adjusting for variables other than adolescents’ mental health status (Models 1, 3, and 5). Compared with individuals who slept for shorter duration, long sleepers had less frequent problem behaviors. Meanwhile, having a sleep disorder was significantly positively correlated with adolescents’ problem behaviors, indicating that sleep disorders significantly increase the frequency of such behaviors.

**TABLE 2:** OLS results before and after adding the mental health variable.

**Variables**	**Y = Total problem behaviors**		**Y = Problem behaviors threaten others**		**Y = Problem behaviors threaten themselves**		***P***
	**Coefficients(SE)**		**Coefficients(SE)**		**Coefficients(SE)**		
	**Model 1**	**Model 2**	**Model 3**	**Model 4**	**Model 5**	**Model 6**	**Model 4 VS 6**
*****Independent variable*****							
Sleep time (Reference category: short sleeper)	–0.347***	–0.210**	–0.205***	–0.107*	–0.142***	–0.103**	0.940
	(0.071)	(0.069)	(0.050)	(0.048)	(0.035)	(0.035)	
Sleep disorders (Reference category: No)	0.593***	0.264***	0.441***	0.204***	0.153***	0.061+	0.002
	(0.066)	(0.065)	(0.042)	(0.044)	(0.036)	(0.035)	
***Mediating variable***							
Mental health status		0.693***		0.499***		0.194***	0.000
		(0.056)		(0.036)		(0.027)	
***Control variables***							
Gender (Reference category: Female)	0.806***	0.862***	0.537***	0.577***	0.269***	0.285***	0.000
	(0.073)	(0.073)	(0.055)	(0.054)	(0.034)	(0.035)	
Ethnicity (Reference category: Minority)	–0.150	–0.139	–0.016	–0.008	–0.134+	–0.131+	0.303
	(0.172)	(0.156)	(0.132)	(0.121)	(0.071)	(0.068)	
Hukou type (Reference category: Non-rural Hukou)	0.061	0.076	–0.003	0.008	0.064	0.068	0.305
	(0.082)	(0.079)	(0.057)	(0.055)	(0.043)	(0.042)	
Resident student (Reference category: No)	0.392**	0.387**	0.283**	0.280**	0.108	0.107	0.050
	(0.131)	(0.127)	(0.087)	(0.083)	(0.071)	(0.071)	
Cognitive ability	–0.237***	–0.235***	–0.072+	–0.071+	–0.165***	–0.164***	0.014
	(0.058)	(0.060)	(0.039)	(0.040)	(0.030)	(0.030)	
Problem behaviors of peers	0.760***	0.710***	0.363***	0.327***	0.397***	0.383***	0.022
	(0.045)	(0.045)	(0.024)	(0.024)	(0.027)	(0.027)	
Academic pressure of Chinese (Reference category: No)	0.179*	0.078	0.099+	0.027	0.079+	0.051	0.664
	(0.082)	(0.077)	(0.058)	(0.054)	(0.040)	(0.040)	
Academic pressure of math (Reference category: No)	0.191*	0.049	0.083	–0.019	0.108*	0.069	0.085
	(0.086)	(0.083)	(0.058)	(0.055)	(0.042)	(0.042)	
Academic pressure of English (Reference category: No)	0.294***	0.198*	0.193***	0.124*	0.101**	0.074*	0.335
	(0.077)	(0.078)	(0.057)	(0.057)	(0.034)	(0.034)	
Often eat junk food (Reference category: No)	0.647***	0.535***	0.451***	0.371***	0.196***	0.165***	0.000
	(0.062)	(0.061)	(0.042)	(0.042)	(0.032)	(0.032)	
Often drink sugary or carbonated beverages (Reference category: No)	0.593***	0.484***	0.424***	0.346***	0.168***	0.138***	0.000
	(0.066)	(0.066)	(0.048)	(0.048)	(0.031)	(0.031)	
**Economic status (Reference category: Economic hardship)**							
Middle class	0.077	0.169+	0.090	0.157*	–0.014	0.012	0.066
	(0.102)	(0.101)	(0.075)	(0.074)	(0.053)	(0.052)	
Wealthy	–0.016	0.125	0.075	0.175+	–0.090	–0.051	0.026
	(0.139)	(0.141)	(0.101)	(0.100)	(0.069)	(0.071)	
Parents married (Reference category: Others)	–0.245+	–0.163	–0.247**	–0.189*	0.003	0.026	0.009
	(0.132)	(0.131)	(0.094)	(0.092)	(0.059)	(0.059)	
An only child (Reference category: No)	–0.345***	–0.302**	–0.229***	–0.199**	–0.115**	–0.104*	0.132
	(0.091)	(0.090)	(0.066)	(0.065)	(0.042)	(0.043)	
**Education expectation (Reference category: High school and below)**							
Universities or colleges graduates	–0.411***	–0.399***	–0.192*	–0.184*	–0.218***	–0.215***	0.734
	(0.099)	(0.094)	(0.077)	(0.073)	(0.058)	(0.058)	
Postgraduate and above	–0.756***	–0.749***	–0.443***	–0.438***	–0.313***	–0.311***	0.167
	(0.122)	(0.117)	(0.088)	(0.084)	(0.063)	(0.063)	
Not to matter	0.285	0.247	0.010	–0.018	0.275*	0.264*	0.088
	(0.243)	(0.245)	(0.168)	(0.171)	(0.121)	(0.121)	
Family members smoke (Reference category: No)	0.265**	0.222**	0.223***	0.192***	0.042	0.030	0.000
	(0.079)	(0.077)	(0.053)	(0.052)	(0.035)	(0.034)	
Constant	6.093***	6.536***	3.665***	3.984***	2.428***	2.552***	
	(0.375)	(0.368)	(0.232)	(0.223)	(0.197)	(0.199)	
Sample size	7,879	7,879	7,879	7,879	7,879	7,879	
R-squared	0.288	0.318	0.198	0.235	0.281	0.291	
Adjusted R-squared	0.286	0.316	0.196	0.233	0.279	0.289	

*** *p* < 0.001, ** *p* < 0.01, * *p* < 0.05, + *p* < 0.1. Models 1, 3, and 5 are regression results without mental health status. Models 2,4, and 6 added mental health status as an independent variable. All standard errors were clustered at school level.

In the models that add adolescents’ mental health status (Models 2, 4, and 6), sleep time and the existence of sleep disorders can also substantially increase the frequency of adolescents’ problem behaviors. Sleep disorders have a more significant effect on adolescents’ problem behaviors that threaten others (tested by the seemingly uncorrelated regression estimation method), after distinguishing the problem behaviors that threaten others and threaten oneself (see Models 4 and 6).

Models 2, 4, and 6 also indicate that mental health status substantially affects adolescents’ problem behaviors, implying that the worse the mental health status, the higher the frequency of such behaviors. Moreover, the test of the seemingly uncorrelated regression estimation method indicates that mental health status has a more significant impact on the problem behaviors of adolescents that threaten others than the problem behaviors that threaten oneself. Moreover, compared with the model that did not include the mental health status of adolescents, the coefficients of adolescents’ sleep time and sleep disorders both changed significantly after adding the mental health status. These results suggest that mental health variables may play an important role in sleep time and sleep disorders that affect problem behaviors. This study further explores the mediating effect to investigate how adolescents’ sleep status affects their problem behaviors through their mental health.

In addition, the study also found that boys had a higher frequency of problem behaviors than girls. In addition, resident students had a higher frequency of problem behaviors than non-resident students. Adolescents with peers who had more problem behaviors exhibited problem behaviors more frequently. Adolescents with poor living habits (eating junk food and drinking sugary or carbonated beverages often) had a more frequent occurrence of problem behaviors than those with healthy living habits. Compared with adolescents with lower cognitive ability, adolescents with higher cognitive ability had a relatively lower frequency of problem behaviors; compared with non-only children, only children had a lower frequency of problem behaviors. Compared with adolescents whose family members did not smoke, those whose family members smoked had a higher frequency of problem behaviors.

Before the mediation analysis, the causal relationship between sleep status and adolescent problem behaviors should be confirmed. Despite explicitly capturing the association between them using the OLS regression, we further use the PSM method to verify causality (see Appendix Table A1).

### Mediation Analysis Results

Table 3 demonstrates the mediation analysis results using the bootstrap method to examine the mediating effect of adolescents’ mental status on problem behavior. The results show that these mediating effects are significant, which indicates that sleep time and sleep disorders can indirectly affect adolescents’ problem behaviors through mental health. Specifically, the increase in sleep time of adolescents is conducive to their mental health, which may reduce the frequency of their problem behaviors, whereas the presence of adolescents’ sleep disorders can be detrimental to their mental health status.

**TABLE 3:** Mediating effect size of mental health status and its proportion in the effect of sleep status on problem behaviors (*N* = 7879).

**Effect**	**Path**	**Effect size**	**Bootstrap SE**	**P-value**	**95% CI**	**Proportion^a^**
Total effect		–0.347	0.069	0.000	–0.483, –0.211	
Mediation effect	Sleep time– > Mental health– > Total problem behaviors	–0.137	0.018	0.000	–0.173, –0.101	39.48%
Total effect		–0.205	0.050	0.000	–0.303, –0.107	
Mediation effect	Sleep time– > Mental health– > Problem behaviors that threaten others	–0.099	0.012	0.000	–0.123, –0.074	48.29%
Total effect		–0.141	0.034	0.000	–0.208, –0.075	
Mediation effect	Sleep time– > Mental health– > Problem behaviors that threaten themselves	-0.038	0.007	0.000	–0.052, –0.025	26.95%
Total effect		0.593	0.065	0.000	0.466, 0.721	
Mediation effect	Sleep disorder– > Mental health – > Total problem behaviors	0.329	0.028	0.000	0.274, 0.385	55.48%
Total effect		0.441	0.041	0.000	0.359, 0.522	
Mediation effect	Sleep disorder– > Mental health– > Problem behaviors that threaten others	0.237	0.019	0.000	0.201, 0.273	53.74%
Total effect		0.153	0.036	0.000	0.082, 0.224	
Mediation effect	Sleep disorder– > Mental health– > Problem behaviors that threaten themselves	0.092	0.013	0.000	0.067, 0.118	60.13%

*(1) a is the proportion of the mediating effect on the total effect. (2) When the bootstrapping method was applied in Stata 15.1, we used the software’s default options for subsample size but set the replications 1000 times.*

The results shown in Table 3 indicate that the mediating effect of mental health accounted for 39.48% of the influence of sleep time on adolescents’ problem behaviors. When distinguishing problem behaviors that threaten others and those that threaten themselves, mental health status that mediates the effects of sleep time on problem behaviors that threaten others accounted for a higher proportion, reaching 48.29%. The mediating effect of mental health accounted for 55.48% of the effects of sleep disorders on adolescents’ problem behaviors. After distinguishing between the problem behaviors that threaten others and the problem behaviors that threaten oneself, the mediating effect of mental health status on the influence of sleep disorders on the problem behaviors that threaten oneself was higher, reaching 60.13%.

There were significant differences in the mediating effects of mental health status on sleep time and sleep disorders on adolescents’ problem behaviors. Among the effects of sleep time on adolescents’ problem behaviors, the mediating effect size of mental health status was –0.137, accounting for 39.48% of the total effect. Among the effects of sleep disorders on adolescents’ problem behaviors, the mediating effect of mental health status was greater, accounting for more than 50% of the total effect. Overall, the mediating effect of adolescents’ mental health status regarding the influence of sleep disorders on their problem behaviors was greater than the mediating effect of their sleep time on their problem behaviors.

## Discussion and Conclusion

The present study examined the relationship between sleep time, sleep disorders, and adolescents’ problem behaviors, and it found that both sleep time and sleep disorders significantly increased the frequency of adolescents’ problem behaviors. By further distinguishing the problem behaviors that threaten others and the problem behaviors that threaten oneself, the study found that the association between sleep disorders and the problem behaviors that threaten others was substantially greater than the correlation with the problem behaviors that threaten oneself. The results of the mediating effect analysis suggest that adolescents’ mental health status plays an important role in mediating the effects of sleep status on problem behaviors. Sleep time and sleep disorders indirectly affect adolescents’ problem behaviors through adolescents’ mental health. Moreover, the mediating effect of adolescents’ mental health status on the influence of sleep disorders on their problem behaviors was seemingly greater than its mediating effect of the influence of sleep time.

This study found that short sleep time and the existence of sleep disorders could significantly increase the frequency of adolescents’ problem behaviors, which is consistent with previous studies (O’Brien and Mindell, 2005; Catrett and Gaultney, 2009; Kamphuis et al., 2012; Peach and Gaultney, 2013; Barnes and Meldrum, 2014; Raine and Venables, 2017). Sleep is a resource in daily life that can make people energetic (Zohar et al., 2005) and can provide opportunities for central nervous system recovery (Kolb et al., 2016). For adolescents, sleep disorders mean that they can still not get adequate and quality sleep, despite having adequate sleep opportunities. Studies have shown that lack of sleep can reduce the metabolic rate and brain activity (Durmer and Dinges, 2005), and the brain’s prefrontal cortex is involved in human cognition, judgment, decision making, and emotional control (Beebe and Gozal, 2002). Failure to fulfill these essential functions will increase the frequency of problem behaviors.

In addition, the study revealed that adolescents’ sleep time and sleep disorders affected their problem behaviors by altering their mental health status. Sufficient sleep time for adolescents is conducive to their mental health, thereby reducing the frequency of their problem behaviors, whereas the existence of adolescents’ sleep disorders would be detrimental to their mental health status, thereby increasing their frequency of problem behaviors. One possible reason is that the adverse effects of insufficient sleep on brain function (especially the frontal cortex function) can impair emotional regulation and arousal (Durmer and Dinges, 2005). Moreover, lack of sleep and insomnia can also adversely affect the interaction of the prefrontal cortex, striae, and amygdala, leading to the formation of negative emotions (Soffer-Dudek et al., 2011; Roberts and Duong, 2013). Therefore, inadequate sleep time and the existence of sleep disorders are not conducive to the mental health status of young people from a physiological point of view. Adolescents’ physical and psychological development is still immature. Furthermore, when facing problems, adolescents with impaired mental health can be more impulsive and lack rational thinking, which may contribute to a higher possibility of problem behaviors.

There are some limitations to this study. It hypothesized that sleep affects adolescents’ problem behaviors, but some endogenous issues between sleep time, sleep disorders, and problem behaviors may exist as well. For instance, there is a two-way causal relationship between sleep status and problem behaviors. In addition, there are some missing variables despite the family characteristics, academic pressure, and living habits controlled in the study. However, some empirical studies have found that there is a causal relationship between the two (Meijer et al., 2010; Beebe, 2011), and we also verified the causality between sleep status and adolescents’ problem behaviors through the propensity score matching method. Moreover, the measurement of sleep disorder was simply a yes or no answer, which cannot capture the sleep disorder severity. If we obtain more detailed data about sleep problems, further research on their severity could be conducted.

Despite these limitations, this study has made some important theoretical contributions. As we previously discussed, the relationship between sleep and problem behaviors has been widely analyzed, but the mechanism has not been well investigated. By introducing mental health as a key mediating variable, this study examined how adolescents’ sleep status affects their mental health status and consequently their problem behaviors. As previously mentioned, the increase in sleep time of adolescents is conducive to their mental health, which may reduce the frequency of their problem behaviors, whereas the presence of adolescents’ sleep disorders can be detrimental to their mental health status, having the opposite effect. In recent decades, attention has been increasingly paid to the exploration of mechanisms in the field of social science research. This study thus enriches and broadens the research in this field, and also provides research perspectives and inspiration for further research.

There are also some policy implications from the findings of this study. The “PRC Law on the Prevention of Juvenile Delinquency (Draft Revisions)” proposes special education for juveniles with extreme problem behaviors, which thoroughly reflects the severity of, and the need to prevent, the consequences of juvenile problem behaviors. This research indicates that the frequency of adolescents’ problem behaviors is not only directly related to their sleep status, but is also influenced by their mental health. Thus, sufficient attention should be paid to the sleep status and mental health of Chinese adolescents. According to the “Management Standards for Compulsory Education Schools” issued by the Ministry of Education in 2017, junior high school students should have at least 9 h of sleep, but the average sleep time for adolescents in this study was approximately 8.01 h, and nearly half (44.40%) had one or more types of sleep disorders. Considering this, publicity on the importance of sleep should be increased at a social level, and widespread research should be conducted on the potential effects of sleep time and sleep quality on life. At the school level, the time to school should be postponed within a reasonable range, and mental health education should also be strengthened. Individual provinces have implemented measures to ensure adequate sleep time for students. For example, on February 24, 2018, the Provincial Department of Education of Heilongjiang released the “Notice on Postponement of the Province’s Early Morning Arrival Time for Primary and Secondary School Students,” and it was decided that the province’s primary and middle school students would arrive no earlier than 8:00 in the morning from the beginning of the new semester on March 1. To ensure the mental health of students, the Mental Health Law of the People’s Republic of China explicitly states that “Schools at all levels and types of schools should provide mental health education to students, allocate or hire mental health education teachers and counselors, and must establish mental health counseling rooms to carry out mental health education to students.” It would be more conducive to reduce the occurrence of adolescents’ problem behaviors by improving both their sleep status and mental health. Finally, parents should guide adolescents at the family level to build healthy living habits, recognize and solve their sleep problems promptly, pay close attention to young people’s mental health, promptly guide young people’s negative emotions, and prevent their problem behaviors from occurring.

## Data Availability Statement

Publicly available datasets were analyzed in this study. This data can be found here: (The China Education Panel Survey) (https://ceps.ruc.edu.cn/index.php?r=index/index).

## Ethics Statement

The studies involving human participants were reviewed and approved by Ethics Committee of Renmin University of China. Written informed consent to participate in this study was provided by the participants’ legal guardian/next of kin.

## Author Contributions

YL chosed and developed the research topic. YL and SZ constructed model, analyzed the data, interpreted the results, drafted the manuscript, and had primary responsibility for review and editing of the final content. YL, WL, and HL critically reviewed drafts of the manuscript. All authors read and approved the final version of the manuscript for publication.

## Conflict of Interest

The authors declare that the research was conducted in the absence of any commercial or financial relationships that could be construed as a potential conflict of interest.

## Publisher’s Note

All claims expressed in this article are solely those of the authors and do not necessarily represent those of their affiliated organizations, or those of the publisher, the editors and the reviewers. Any product that may be evaluated in this article, or claim that may be made by its manufacturer, is not guaranteed or endorsed by the publisher.

## Footnotes

1 Common sleep disorders are classified into three groups according to the third edition of the *International Classification of Sleep Disorders* (ICSD-III) released by the American Academy of Sleep Medicine (AASM) in 2014: “insomnia,” “parasomnia,” and “other sleep disorders.” Specific symptoms included difficulty in falling asleep, easy to wake up, fatigue after waking up, sleepwalking, sleep talking, snoring, lethargy, and teeth grinding.

2 CEPS has designed a set of cognitive ability test questions for eighth-grade students. The content of these question does not involve the specific memorable knowledge taught in the school curriculum, but it measures students’ logical thinking and problem-solving ability. Scores can be compared with international and national standards. The standardized scores of cognitive ability were used in the analysis.

3 Peer misconduct was measured by the following questions: Do you have any good friends who have been truant? Do you have any good friends who are criticized or punished for violation of school discipline? Do you have any good friends who fight with others? Do you have any good friends who smoke or drink? Do you have any good friends who often surf the Internet or go to game halls? The answer to each question was selected from three possible responses: “no such friends,” “1 or 2,” and “many friends.” The values were assigned to 1, 2, 3 in turn. The total score was determined by calculating the score on five questions. The higher the score, the greater probability the problem behaviors adolescents’ peers have.

4 From the CEPS questionnaire, we used the question “Do any of the family members smoke?” to measure whether there are any family members smoking or not. It was a dummy variable.

## Appendix

**TABLE A1:** Main results of the propensity score matching analysis.

	**Total problem behaviors**			**Problem behaviors threaten others**			**Problem behaviors threaten themselves**		
	
	**Treated**		**Controls**	**Treated**		**Controls**	**Treated**		**Controls**
***Panel A***									
**Sleep time**									
(1) ATT	11.311		11.532	6.476		6.599	4.835		4.933
(2) SE		0.092			0.060			0.044	
(3) t		–2.41			-2.07			–2.22	
**Sleep disorders**									
(1) ATT	11.870		11.577	6.857		6.640	5.012		4.937
(2) SE		0.091			0.059			0.044	
(3) t		3.23			3.68			1.72	

***Panel B***									
**Sleep time**									
(1) ATT	11.311		11.530	6.476		6.601	4.835		4.929
(2) SE		0.091			0.059			0.044	
(3) t		–2.41			-2.12			–2.15	
**Sleep disorders**									
(1) ATT	11.881		11.584	6.865		6.642	5.016		4.942
(2) SE		0.089			0.058			0.043	
(3) t		3.33			3.83			1.72	

***Panel C***									
**Sleep time**									
(1) ATT	11.311		11.533	6.476		6.596	4.835		4.937
(2) SE		0.096			0.062			0.046	
(3) t		–2.31			–1.92			–2.21	
**Sleep disorders**									
(1) ATT	11.881		11.587	6.865		6.662	5.016		4.925
(2) SE		0.094			0.061			0.045	
(3) t		3.13			3.31			2.02	

*Panel A was estimated using the radius-matching method (0.01). Panel B was estimated using the kernel-matching method (0.06). Panel 3 was estimated using the k-nearest-neighbor-matching method.*
